# ANCHOR-Grid: Authenticating Smart Grid Digital Twins Using Real-World Anchors

**DOI:** 10.3390/s25102969

**Published:** 2025-05-08

**Authors:** Mohsen Hatami, Qian Qu, Yu Chen, Javad Mohammadi, Erik Blasch, Erika Ardiles-Cruz

**Affiliations:** 1 Department of Electrical and Computer Engineering, Binghamton University, Binghamton, NY 13902, USA; mhatami1@binghamton.edu (M.H.); or qqu@vsu.edu (Q.Q.); 2 Department of Computer Science, Virginia State University, Petersburg, VA 23806, USA; 3 Department of Civil, Architectural, and Environmental Engineering, The University of Texas at Austin, Austin, TX 78705, USA; javadm@utexas.edu; 4 The U.S. Air Force Research Laboratory, Rome, NY 13441, USA; erik.blasch.1@us.af.mil (E.B.); erika.ardiles-cruz@us.af.mil (E.A.-C.)

**Keywords:** Internet of Smart Grid Things (IoSGT), Deepfake attacks, digital twins (DTs), Electrical Network Frequency (ENF) signal, environmental fingerprints

## Abstract

Integrating digital twins (DTs) into smart grid systems within the Internet of Smart Grid Things (IoSGT) ecosystem brings novel opportunities but also security challenges. Specifically, advanced machine learning (ML)-based Deepfake technologies enable adversaries to create highly realistic yet fraudulent DTs, threatening critical infrastructures’ reliability, safety, and integrity. In this paper, we introduce Authenticating Networked Computerized Handling of Representations for Smart Grid security (ANCHOR-Grid), an innovative authentication framework that leverages Electric Network Frequency (ENF) signals as real-world anchors to secure smart grid DTs at the frontier against Deepfake attacks. By capturing distinctive ENF variations from physical grid components and embedding these environmental fingerprints into their digital counterparts, ANCHOR-Grid provides a robust mechanism to ensure the authenticity and trustworthiness of virtual representations. We conducted comprehensive simulations and experiments within a virtual smart grid environment to evaluate ANCHOR-Grid. We crafted both authentic and Deepfake DTs of grid components, with the latter attempting to mimic legitimate behavior but lacking correct ENF signatures. Our results show that ANCHOR-Grid effectively differentiates between authentic and fraudulent DTs, demonstrating its potential as a reliable security layer for smart grid systems operating in the IoSGT ecosystem. In our virtual smart grid simulations, ANCHOR-Grid achieved a detection rate of 99.8% with only 0.2% false positives for Deepfake DTs at a sparse attack rate (1 forged packet per 500 legitimate packets). At a higher attack frequency (1 forged packet per 50 legitimate packets), it maintained a robust 97.5% detection rate with 1.5% false positives. Against replay attacks, it detected 94% of 5 s-old signatures and 98.5% of 120 s-old signatures. Even with 5% injected noise, detection remained at 96.5% (dropping to 88% at 20% noise), and under network latencies from <5 ms to 200 ms, accuracy ranged from 99.9% down to 95%. These results demonstrate ANCHOR-Grid’s high reliability and practical viability for securing smart grid DTs. These findings highlight the importance of integrating real-world environmental data into authentication processes for critical infrastructure and lay the foundation for future research on leveraging physical world cues to secure digital ecosystems.

## 1. Introduction

The Metaverse has attracted a lot of attention [1,2,3]. As a virtual extension of our social and professional lives, the Metaverse redefines how we live, work, and socialize by enabling seamless interweaving of the physical world with a virtual cyberspace [4]. The smart grid can benefit from the Metaverse by using its virtual environments for simulation, training, and collaboration. For example, energy providers can leverage the Metaverse to visualize grid performance, optimize energy distribution, or train operators in realistic, risk-free virtual scenarios, enhancing grid reliability and sustainability [5]. Meanwhile, there have also been increased concerns about identity security and authenticity [6]. For example, advanced Deepfake technologies allow malicious actors to create highly realistic but fraudulent data, posing significant risks such as data theft, misinformation, and erosion of trust within virtual communities [7]. These risks can have severe consequences in sensitive applications such as the smart grid.

Technologies such as blockchains [8], Elliptic Curve Cryptography (ECC) [9], evidential reasoning [10], and fuzzy logic [11] are introduced to secure access control for the Metaverse and to perform cross-domain ID authentication. However, even with all these advances, substantial work still needs to be carried out to secure sensitive information for devices to interact in the virtual world, due to the lack of attention paid in the past. Currently, security mechanisms in the Metaverse are in their infancy, making the Metaverse vulnerable to impersonation attacks and data theft.

Security is a crucial aspect of modern power infrastructure, integrating advanced information technology to improve efficiency, reliability, and sustainability [12,13]. A smart power grid is considered part of the future Metaverse, relying on interconnected devices such as sensors, smart meters, and automated control systems that collect and share data to optimize energy distribution and consumption [14]. However, this increased connectivity also presents vulnerabilities to cyberattacks, data breaches, and system manipulation, which can severely affect the stability and public safety of the network [15].

The concept of Deepfake attacks on the smart grid introduces significant risks, as the grid relies heavily on digital communication, automation, and artificial intelligence (AI) for efficient operation [16]. Deepfake attacks in this context involve generating or altering data, signals, or interactions to mislead systems or operators, potentially compromising the stability and security of the grid. The consequences of such attacks can range from operational disruptions to financial losses and even national security threats [17]. One potential scenario is the generation of fake control signals or the manipulation of legitimate ones. These Deepfake signals could mislead operators or automated systems, leading to load-balancing failures, unnecessary load shedding, or even widespread grid outages [18]. Similarly, Deepfake technology can manipulate sensor data, creating synthetic or altered measurements of voltage, current, or frequency [19].

Such manipulation could mislead AI systems used for predictive maintenance, trigger false alarms, or conceal actual system faults, leading to inefficiencies and vulnerabilities [20]. Another form of Deepfake attack involves impersonating grid operators or stakeholders. Through advanced video or voice Deepfake technology, attackers can issue false commands or mislead participants in decision-making processes [21]. A voice attack could result in unauthorized actions, such as shutting down critical infrastructure or overloading the network.

Deepfake attacks could also target consumer data, fabricating electricity-consumption patterns to create incorrect billing or disrupt demand-side management programs [22]. Furthermore, attackers might exploit biometric authentication systems, such as facial or voice recognition, used in grid-control systems or customer portals [23]. By bypassing authentication mechanisms, they could gain unauthorized access to critical infrastructure or steal sensitive data. The consequences of these attacks are severe. False data or signals could disrupt power generation, transmission, and distribution operations, leading to cascading failures across interconnected infrastructures [24]. In addition, Deepfake attacks could undermine trust in the smart grid as users and stakeholders struggle to distinguish between genuine and fake communications, ultimately increasing vulnerability and risking national security [25].

Advanced technologies like Electric Network Frequency (ENF)-based security [26], blockchain [27], DTs [28], and AI-driven threat detection [29] are increasingly essential to improve the security of the smart grid. ENF can ensure authenticity by anchoring communications to an unpredictable signal in the real world. DTs are a virtual representation of the infrastructure, such as computer-aided designs of buildings, bridges, and vehicles [30]. The DT can be used to cross-check the functioning of applications when information comes from distributed sensors. This paper proposes Authenticating Networked Computerized Handling of Representations for Smart Grid Security (ANCHOR-Grid), a novel method for authenticating smart grid DTs by leveraging ENF signals as anchors in the real world.

The primary contributions of this paper include:

- *Expansion of Environmental Fingerprinting into Virtual Worlds.* Pioneering application in the Metaverse, our work extends the concept of environmental fingerprinting beyond physical and network security to virtual DT environments, an unexplored area.
- *Protection of Critical Infrastructure.* Contributing to smart grid security by proposing a novel authentication mechanism that bridges the physical and virtual worlds and addresses a critical gap in existing security measures against Deepfake attacks in critical infrastructure systems.
- *Real-World Validation via Smart Grid Case Study.* We validated the proposed ANCHOR-Grid in a virtual IoSGT environment, demonstrating its ability to distinguish authentic DTs from Deepfakes using ENF signatures and its practicality and scalability for smart grid security.

The remainder of this paper is structured as follows. Section 2 provides a brief overview of current state-of-the-art technology and background knowledge. Section 3 details the principle, architecture, and algorithm of the ANCHOR-Grid system. Section 4 validates the effectiveness and presents the performance of ANCHOR-Grid using the case study. Section 5 describes the experimental setup, evaluation methodology, key results that demonstrate detection rates, robustness under noise and network conditions, and comparison with baseline methods. Finally, Section 6 concludes this paper with conclusions and a concise discussion of ongoing efforts.

## 2. Background and Related Works

### 2.1. Data Security in the Metaverse

As the adoption of DT and the Metaverse expands in smart grid environments, ensuring the security and authenticity of virtual representations becomes a critical challenge [31]. Although traditional power grid security mechanisms focus on load frequency control (LFC) and protection against physical cyber attacks [32], integrating DTs and the Metaverse requires different security strategies that safeguard data integrity, network communication, and authentication mechanisms in virtualized environments. Recent studies have proposed blockchain-based authentication systems for DTs, leveraging decentralized verification to prevent tampering and unauthorized modifications [33]. For example, research on blockchain-enhanced DT security has demonstrated the potential to use distributed ledgers to ensure digital assets’ integrity in virtual power systems [34]. However, while blockchain can provide tamper-proof authentication, it does not inherently protect against Deepfake-based attacks, where adversaries inject synthetically generated DTs or manipulated sensor data into the system [35]. Additionally, intrusion-detection systems (IDSs) and machine learning-based anomaly detection have been explored to identify cyber threats in smart grids [36]. While these techniques can detect anomalous network behavior or malicious access attempts, they do not provide real-time authentication of DTs or multimedia-based representations in the Metaverse.

The current data security landscape for the Metaverse covers various aspects such as user identification, avatar protection, data authentication, etc. Different techniques are introduced, such as cryptography, blockchain, machine learning, and biometric-based approaches. For example, to secure the biometric data-transmission process for the Metaverse, ECC and fuzzy extractors are combined to achieve lower computational and transmission usage while maintaining similar robustness against specific attacks [9]. Similarly, a framework using chameleon signature authentication was proposed to allow users to obtain ID tokens from third-party providers using biometric information [37]. The scheme continuously verifies the identities by connecting the users’ real IDs with the avatars’ virtual IDs. Although this approach guarantees authentication reliability, adopting the Chameleon algorithm creates additional processing time. In recent years, machine learning has been a practical technique to enhance the current security landscape of the power grid, especially in SCADA (supervisory control and data acquisition) systems [38].

As smart grids transition into Metaverse-integrated environments, they become vulnerable to Deepfake attacks, where adversaries attempt to create and inject fake DTs that do not correspond to real-world grid components [39]. These malicious DTs can disrupt grid operations, manipulate system parameters, or mislead operators into making erroneous decisions. In a worst-case scenario, an attacker could introduce a fraudulent DT of a critical substation that falsely reports normal operating conditions. At the same time, a physical outage or security breach occurs, leading to delayed response times and potential widespread failures [40]. Deepfake DT attacks pose a significant challenge because visual similarity and software-based validation alone cannot distinguish authentic DTs from fraudulent ones in a purely virtual environment like the Metaverse [39]. Attackers can employ AI-generated synthetic data, historical replay attacks, or cloned DT models to trick conventional security mechanisms. This is where ANCHOR-Grid’s ENF-based authentication plays a crucial role in ensuring that each DT remains verifiably linked to its physical counterpart.

By embedding ENF signals, real-time frequency variations unique to each grid interconnect, into the DT data stream, ANCHOR-Grid prevents fake DTs from mimicking legitimate components. Since an attacker cannot predict or manipulate ENF variations in real-time without direct access to the physical grid, any fraudulent DT lacking a valid ENF signature is immediately flagged as inauthentic. Additionally, our blockchain-based validation system cross-verifies ENF timestamps across multiple DTs, making it impossible for an adversary to forge consistent ENF data across the entire system. Even if a Deepfake DT manages to bypass initial software-based authentication, its inability to present valid, real-time ENF signatures results in its rejection from the Metaverse-integrated smart grid.

### 2.2. ENF Signals as an Environmental Fingerprint

**Figure 1:**
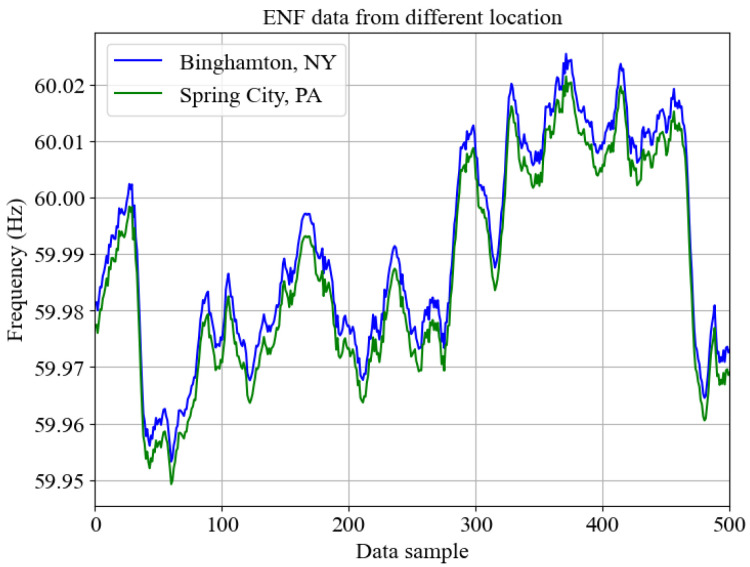
ENF signals simultaneously collected from two cities.

ENF is a time-varying signal fluctuating across its nominal frequency value of 50 Hz or 60 Hz based on the power supply demand from electrical power grids [26]. In the United States, the ENF nominal value is 60 Hz, whereas most Asian and European countries have a nominal value of 50 Hz. Due to the power supply and demand fluctuations throughout the power grid, the load balance mechanism of the generator system results in the fluctuation of ENF from its nominal value within a range. The ENF fluctuates in the range of [−0.02, 0.02] Hz in the United States and [−0.05, 0.03) Hz in Asian and European countries [41]. The fluctuations in the ENF signal are propagated throughout the power grid interconnect, and thereby, any observed ENF fluctuations for a particular time instant should reflect similar volatility. Figure 1 represents the ENF signal simultaneously recorded from two cities in different states, 180 miles apart, where both cities belong to the Eastern power grid interconnect. With ENF estimated from the multimedia recordings and the existence of a parallel ground truth reference database, a signal can be matched for its accurate recording time [42]. Leveraging the unique fluctuating nature and presence in multimedia recordings, the ENF signals have been applied in areas like digital forensics [26], location-based geo-tagging [43], surveillance video authentication [44], and Deepfake attack detection [17].

### 2.3. Digital Twins in Smart Grids

As the technology refers to creating a virtual representation of a physical object, DTs can mirror the characteristics, behaviors, and conditions in real-time [3]. Dynamic DTs are utilized with static DTs using the Dynamic Data Driven Application Systems (DDDAS) paradigm [45]. DTs in smart grids create a dynamic, real-time digital replica of the physical, electrical grid, enabling enhanced monitoring, analysis, and optimization of grid operations [46]. DTs are comprehensive virtual models that mirror the actual grid components’ behavior, conditions, and structure, such as transformers, substations, and distribution lines [47]. The DT virtual representation allows utility operators to visualize and understand the grid’s performance under various conditions without interfering with the physical infrastructure.

By implementing DTs, smart grid operators can perform advanced simulations and predictive analytics to anticipate and mitigate potential issues before they escalate into critical problems [48,49]. For example, DTs can model the impact of integrating renewable energy sources such as solar or wind power, forecast demand fluctuations, and assess the effects of equipment failures or cyberattacks. The proactive approach of DT facilitates better decision-making, improves grid reliability, and optimizes energy storage systems (ESS) and distribution by allowing operators to test scenarios and strategies in a virtual environment without risk [50]. Furthermore, DTs play a crucial role in asset management and maintenance within Smart Grids [51]. They enable real-time monitoring of equipment health and performance by collecting and analyzing data from sensors embedded in the grid infrastructure. DTs with continuous data flow allow for predictive maintenance strategies, where potential failures are identified and addressed before they occur, reducing downtime and maintenance costs [52]. Additionally, DTs support the efficient planning and execution of grid expansions or upgrades by providing detailed insights into the existing infrastructure’s capabilities and limitations.

DTs also contribute to the improvement of the cybersecurity of Smart Grids [53]. The risk of cyber threats increases as the energy sector becomes increasingly digitized. DTs can simulate potential cyberattack scenarios and help develop robust defense mechanisms by identifying vulnerabilities within the digital ecosystem of the grid [54]. A DT simulation capability ensures that security measures are effective and up-to-date, protecting the network against disruptions that could have widespread consequences. In the broader context of transitioning to more sustainable and resilient energy systems, DTs facilitate the integration of distributed energy resources and advanced technologies such as the Internet of Things (IoT) devices and smart meters [55,56]. DTs provide a platform for testing the interoperability and impact of these technologies on grid stability and efficiency. By enabling a deeper understanding of how different components interact within the grid, DTs support the evolution of smart grids toward greater adaptability, sustainability, and customer-centric services.

## 3. ANCHOR-Grid: Rationale and Design

### 3.1. Architecture Overview

Integrating DTs and the Metaverse is poised to revolutionize smart grid operations by enabling real-time monitoring, control, and predictive analysis in a secure, virtual environment. DTs are virtual representations of physical components of the smart grid, such as transformers, substations, power lines, and renewable energy sources. These twins allow operators to analyze grid performance, conduct simulations, and implement security mechanisms without directly interacting with the physical infrastructure. When integrated into the Metaverse, DTs become highly immersive and interactive, allowing engineers and security teams to monitor and respond to grid events with enhanced situational awareness.

However, with this increased virtualization comes new security threats, particularly from Deepfake DTs and adversarial manipulations within the Metaverse. Unlike traditional cybersecurity threats, attacks on a Metaverse-integrated smart grid can involve forged virtual components, manipulated real-time data, and unauthorized control over grid assets. This requires an authentication mechanism that ensures that each DT is genuinely linked to its real-world counterpart and cannot be spoofed or altered by adversaries. Our ANCHOR-Grid framework addresses this challenge by using ENF signals as environmental fingerprints, ensuring that each DT’s data stream is verifiably tied to a real, physical grid component. By embedding real-time ENF data into the DTs, our framework prevents unauthorized entities from injecting fraudulent or manipulated DTs into the Metaverse. This security layer guarantees that only authentic, grid-connected components are represented in the virtual environment, significantly enhancing the integrity and reliability of Metaverse-integrated smart grids.

**Figure 2:**
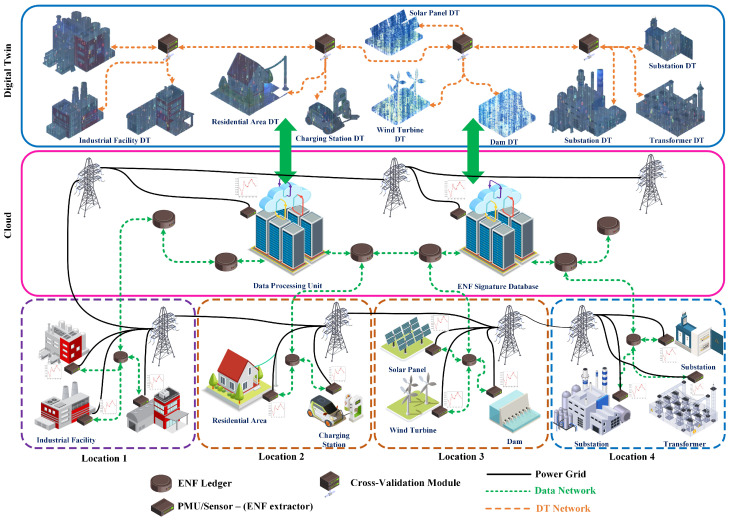
An architectural view of ANCHOR-Grid for virtual health monitoring.

Figure 2 illustrates the architecture of ANCHOR-Grid, showing how ENF signals, DTs, and blockchain-based validation collaboratively secure the smart grid. Each physical component of the smart grid, such as substations, solar panels, and wind turbines, extracts real-time ENF signals and transmits them to its corresponding digital twin (DT). These ENF signals, which fluctuate unpredictably based on grid load variations, serve as real-world anchors, confirming the authenticity of the data streams from physical devices.

The bottom of Figure 2 represents multiple geographical locations connected to the power grid. Each area is part of the smart grid infrastructure and has distinct characteristics.

- Location 1 is an industrial environment with various facilities connected to the power grid (black solid lines). The data network links each facility to the cloud, allowing data aggregation, including ENF readings from this location. The ENF data are a unique identifier consistent in all locations connected to the same power grid, making it a reliable feature to detect data manipulations.
- Location 2 represents a residential area, including homes and electric vehicle charging stations. The ENF signature captured here provides a unique, location-specific electrical frequency profile that can be cross-referenced with data from other locations for consistency. The ENF signal helps verify the authenticity of the data and prevent malicious Deepfake attacks.
- Location 3 shows a specialized industrial and research facility. This location integrates advanced facilities highly dependent on smart grid technologies, and ENF anchors can help ensure that data from this sensitive location are secure. Any inconsistencies in the ENF signal can indicate potential Deepfake attempts or tampering.

The Cloud Layer is a centralized hub that processes the data gathered from distributed locations and manages the DTs. The cloud layer facilitates centralized data processing, analytics, and secure data storage. The cloud architecture ensures that the real-time data captured from physical locations is securely aggregated and cross-referenced with the corresponding digital twin data to identify discrepancies or potential Deepfake attacks. The green dashed lines represent data communication between the cloud and various locations, emphasizing the role of the cloud as a central node that enables synchronization and validation between locations.

The Digital Twin Layer at the top illustrates several DTs of physical infrastructure within the smart grid. DTs are virtual replicas that simulate the behavior and operations of the corresponding real-world assets. These data DTs interact and exchange information through a DT Network (orange dashed lines). Each digital twin replicates a specific element of the smart grid, such as industrial systems, residential environments, or specialized facilities. The primary role of these DTs is to facilitate continuous monitoring, predictive maintenance, and optimization of grid operations. DTs rely on real-time data to accurately mirror physical systems and help cross-verify data authenticity by leveraging ENF anchors as a security measure.

The Power Grid links all three locations to the central grid. This shared power grid means that the ENF signal remains consistent across all locations. Any data manipulation through Deepfake attacks would disturb the expected ENF pattern, which can be used to detect and mitigate the attack. The Data Network connects each location to the cloud, ensuring that operational data, ENF readings, and other relevant information are continuously transmitted for processing and validation. The cloud can use operational information to cross-check data across locations to identify discrepancies. Finally, the DT Network connects the DTs in the top layer, allowing virtual models to interact and exchange information based on the data collected from physical systems. Using ENF as an anchor, these DTs can verify that their data for simulation and decision-making are genuine and free from manipulation.

Once received, each DT verifies its ENF data against a blockchain-based validation system. This step is critical because the blockchain ensures that no single entity can modify or manipulate authentication records, creating a decentralized, tamper-resistant mechanism for DT validation. The blockchain ledger maintains a historical record of ENF signatures, making it impossible for attackers to reuse or replay previous ENF signals without detection. Furthermore, this system allows for cross-validation among multiple DT nodes, meaning that each DT can verify its authenticity against its own ENF data and neighboring DTs within the network.

By integrating ENF, DTs, and blockchain, the ANCHOR-Grid framework creates a multi-layered security structure that protects smart grids from digital impersonation attacks. The combination of real-world ENF fingerprints, immutable blockchain records, and cross-validation among DTs makes it extremely difficult for adversaries to inject fake DTs into the system or manipulate data streams within the Metaverse. This ensures that only authentic, grid-connected DTs exist in the virtual environment, safeguarding the smart grid’s reliability, security, and operational stability. Our study introduces ENF as a unique real-world signature to address this gap by enabling real-time authentication of DT data and securing its integrity within Metaverse smart grid environments.

### 3.2. Rationale of ANCHOR-Grid

The rationale for ANCHOR-Grid lies in the increasing complexity and interconnectivity of smart grid systems, where DT and data integrity, confidentiality, authenticity, and trust are paramount. Integrating DTs has become vital to monitoring, predicting, and optimizing grid performance in modern power grids. However, this digital transformation also presents new challenges in ensuring that the data flowing between physical components and their digital counterparts is authentic and untampered with. Without robust verification mechanisms, malicious actors could inject false data streams or even generate Deepfake data, leading to disruptions, compromised reliability, and even safety hazards in critical energy infrastructure. ANCHOR-Grid addresses these challenges by leveraging ENF signals as a real-world anchor, ensuring that data transmitted to DTs is genuinely from grid-connected sources and is intrinsically tied to real-time conditions.

ENF offers a unique, constantly fluctuating signal synchronized across the power grid, making it an ideal candidate for real-world authentication. Unlike conventional digital signatures or encryption schemes that depend on pre-shared cryptographic keys or trusted third parties, ENF signals are inherently shared across all grid-connected devices in real-time. These fluctuations are unpredictable and difficult to forge without direct access to the grid, making ENF an ideal natural fingerprint for verifying the origin of data. By embedding ENF data into communications between physical devices and their DTs, ANCHOR-Grid provides a reliable, cost-effective, and tamper-resistant method for establishing authenticity, particularly beneficial in distributed smart grid systems requiring real-time responses and decentralized control.

ANCHOR-Grid also provides a means to address synchronization without reliance on traditional mechanisms such as GPS or external time servers, which are vulnerable to spoofing or jamming attacks [57]. The ENF value is automatically synchronized throughout the power grid, eliminating the need for additional hardware or infrastructure to synchronize. The ANCHOR-Grid approach simplifies authentication, reduces deployment costs, and mitigates dependencies on external timing mechanisms. By using ENF as an environmental anchor, ANCHOR-Grid ensures that data are tied to real-world conditions and builds resilience against Deepfake attacks and replay attacks, thus enhancing the robustness of the smart grid’s digital ecosystem. Incorporating ENF-based authentication into the smart grid provides a strong foundation to improve grid reliability, enabling the secure integration of renewable energy sources, and supporting the next generation of digital grid management technologies.

### 3.3. ENF-Based Authentication Module

The exponential growth of interconnected sensor networks in modern technological ecosystems requires robust, lightweight authentication mechanisms. The ENF signatures as an environmental authentication method provide many advantages by taking advantage of their unique frequency fluctuations, consistent throughout the local interconnected grid [58]. Using the sensors deployed for senior safety monitoring, including multimedia-based sensors such as audio or video, can enable ENF embedding as part of the captured recording and provide location- and temporal-based signatures. A dedicated ENF capture sensor can also be deployed without alternative sources, providing redundancy to the authentication framework.

Leveraging lightweight signal estimation algorithms such as the short-time Fourier transform and the correlation coefficient for signal similarity, we establish an ENF authentication module for the data captured from independent sources simultaneously instant [44]. Although the ENF signal is accessible to external adversarial actors, combining the ENF signal and a unique device identifier enables an additional layer of security. Given this measure, remote manipulation of the sensor data becomes challenging due to the location-based fluctuations of the ENF signal and unique sensor device identifiers.

## 4. Security Monitoring for Smart Grid

Instead of a full-scale Metaverse, a task-oriented Microverse allows a more feasible paradigm to create a proof-of-concept prototype [59]. To validate the feasibility and effectiveness of our ANCHOR-Grid scheme, we conducted a case study in a Microverse-based IoSGT environment designed to monitor the security and robustness of a Microgrid.

### 4.1. Attack Scenarios

While cybersecurity threats in smart grids are diverse, including Denial-of-Service (DoS)/Distributed Denial-of-Service (DDoS) attacks, data injection, sensor spoofing, and others, ANCHOR-Grid is specifically designed to counter AI/ML-enabled Deepfake attacks. Unlike traditional cyber-attacks that disrupt network communication or block data flow [60,61], Deepfake attacks involve the synthetic generation of fake data, which poses a unique and growing challenge to DT-based and Metaverse-integrated systems. The Metaverse relies on multimedia, digital avatars, and immersive simulations, all of which are vulnerable to Deepfake manipulations. Attackers can use AI-generated DTs that mimic legitimate components of the smart grid, leading to misinformation, incorrect operational decisions, and potential system failures. Traditional authentication mechanisms, such as cryptographic keys or rule-based anomaly detection, cannot distinguish between a real DT and a fake counterpart. Regarding DoS attacks, ANCHOR-Grid Does Not Focus on them. DoS attacks operate at the network level to disrupt communications rather than falsify data authenticity. Such attacks can be mitigated using conventional network security techniques, such as firewall rules, traffic filtering, and network slicing. ANCHOR-Grid is designed not to address network availability issues but to ensure the authenticity of DTs in the Metaverse, making Deepfake detection its primary objective.

As integrating DTs into the Metaverse becomes more widespread in smart grid monitoring and control, cyber threats such as fake DT injections, replay attacks, and Deepfake-based manipulations pose significant security risks. Attackers can exploit the virtual representation of grid components to manipulate operational data, mislead grid operators, or even gain unauthorized control over smart grid infrastructure. ANCHOR-Grid addresses these challenges by leveraging real-world ENF signals as unique authentication markers, ensuring that only legitimate DTs directly linked to physical grid components can operate within the system. The following outlines three concrete attack scenarios and how ANCHOR-Grid effectively mitigates them.

#### 4.1.1. Scenario 1: Injecting a Fake DT of a Power Substation

In this scenario, an attacker creates a fraudulent DT of a power substation and injects it into the Metaverse-based smart grid monitoring system. This fake DT reports normal operational data, deceiving operators into believing that the substation is functioning correctly, even when experiencing an overload, equipment failure, or security breach. Such an attack could lead to delayed response times, incorrect decision-making, and grid instability or cascading failures. ANCHOR-Grid can prevent it by:

1. Real-Time ENF Verification: The fake DT lacks real-time ENF signals because the attacker cannot generate valid frequency variations that align with the actual fluctuations of the grid. ANCHOR-Grid’s ENF verification module detects anomalies in the DT’s reported frequency signature, immediately flagging it as a non-authentic entity.
2. Blockchain-Based Cross-Validation: The blockchain-based authentication layer further ensures that every DT’s ENF signature is validated across multiple nodes, making it impossible for a fake DT to pass authentication. Since the attacker cannot match the expected ENF sequence from the legitimate power substation, the fraudulent DT is automatically rejected.

By ensuring every DT carries a verifiable real-world ENF signature, ANCHOR-Grid prevents attackers from injecting fake grid components into the Metaverse, protecting smart grid operators from misinformation and operational risks.

#### 4.1.2. Scenario 2: Replay Attack Using Historical ENF Data

A more sophisticated attacker captures previously recorded ENF signals from a valid DT and attempts to replay them later to authenticate a fraudulent DT. This attack could allow the adversary to gain unauthorized control of a critical grid component in the Metaverse, posing serious security risks, including operational disruptions and system manipulation. ANCHOR-Grid prevents this attack by:

1. Time-Sensitive ENF Authentication: ENF signals are highly time-sensitive; each time-stamped signal correlates to a specific moment in real-world grid conditions. If an attacker replays an ENF signature from an earlier time, it will not match the real-time fluctuations occurring in the grid, leading to immediate detection.
2. Blockchain-Recorded ENF Trend Comparison: ANCHOR-Grid compares the incoming ENF data with the trend recorded on the blockchain for ENF. Since historical ENF data do not align with real-time variations, the system automatically detects the mismatch and rejects the replayed DT.
3. Timestamp-to-ENF Correlation Analysis: If the timestamp of the replayed attack data does not align with the expected ENF signature, the system flags the DT as inauthentic and denies access.

By integrating time-sensitive ENF authentication and blockchain validation, ANCHOR-Grid prevents replay attacks and ensures that every DT’s identity remains dynamically linked to its real-world counterpart.

#### 4.1.3. Scenario 3: Deepfake DTs Generating Synthetic Data

One of the most advanced threats to Metaverse-integrated smart grids involves using AI-generated Deepfake DTs. Attackers can use sophisticated machine learning models to create synthetic yet highly realistic DTs that mimic legitimate smart grid components. These DTs may generate convincing but fake operational data, deceiving monitoring systems into accepting falsified sensor readings, power flow information, or grid conditions. ANCHOR-Grid prevents this scenario by:

1. Inability to Replicate Real-Time ENF Fluctuations: While AI-generated Deepfake DTs can simulate sensor outputs and operational patterns, they cannot replicate real-time ENF fluctuations, which are intrinsically tied to the physical grid. Any DT lacking a real-time ENF correlation is automatically flagged as suspicious.
2. Correlation Threshold-Based Authentication: ANCHOR-Grid sets a strict ENF correlation threshold (0.8), ensuring that only genuine ENF data extracted in real-time passes authentication. If a Deepfake DT fails to align with actual grid conditions, it is immediately rejected from the system.
3. Cross-Validation with Physical Grid Data: The system cross-checks the DT’s reported ENF signature against real-world smart grid fluctuations. Since Deepfake DTs cannot interact with physical power systems, they lack the correct ENF fingerprint and fail authentication.

We empirically selected a Pearson correlation cutoff of 0.8 between client and server ENF streams because genuine ENF pairs consistently exceeded 0.9 under up to 5% noise and 200 ms latency, while replayed or Deepfake ENF segments never surpassed 0.7. The threshold of 0.8 sits at the Correlation Coefficient curve’s elbow, yielding over 97% attack detection and under 1.5% false positives across Deepfake and replay scenarios. A higher threshold would reject legitimate readings with minor jitter, and a lower threshold would admit some fakes. Thus, 0.8 offers a practical balance between tolerance for real-world imperfections and strictness against manipulated signals.

### 4.2. ANCHOR-Grid Microverse

In this work, we harness Unreal Engine 5 (UE5) to pioneer a real-time monitoring environment for Microgrid DTs, seamlessly integrating sensor data and attack event simulations to enhance smart grid security and resilience. Using advanced 3D modeling, rendering, and visualization tools from UE5, we construct a highly realistic and interactive virtual representation of the Microgrid infrastructure, embedding dynamic data visualization, ENF-based authentication, and simulated Deepfake attack scenarios. This implementation showcases the power of the Microverse as a cutting-edge platform for DT simulation. It establishes a robust framework for real-time security analysis, offering unparalleled immersion and actionable insights into microgrid operations under normal and adversarial conditions.

Based on live or pre-recorded data, real-time sensor integration is achieved through UE5’s Blueprint Visual Scripting, enabling dynamic updates to component statuses, such as temperature, voltage, and power usage. This flexibility ensures adaptability to diverse operational contexts, with UE5 facilitating API-driven real-time data feeds or simulated datasets processed by a server and visualized in the virtual environment. The result is a responsive, data-driven simulation that mirrors physical grid dynamics with high fidelity, providing a practical tool for monitoring and securing critical infrastructure.

**Figure 3:**
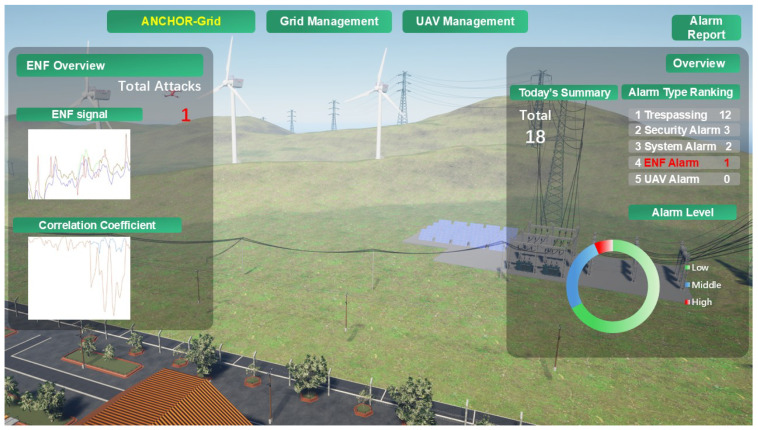
Real-time GUI of the virtual smart grid in Unreal Enginvb 5.

As illustrated in Figure 3, our virtual smart grid scenario encapsulates key elements of a Microgrid, including a local substation, solar panels, wind turbines, transmission lines, monitoring UAVs, and a small factory. Each of them is meticulously modeled to reflect real-world counterparts. By integrating live sensor data and physical facility information, this virtual space accurately replicates the operational state of the smart grid, enabling real-time management of power distribution and UAV coordination [62]. A notable feature is the daily alarm report, which categorizes events such as trespassing, security breaches, ENF anomalies, and UAV-detected issues. The ENF alarm, in particular, leverages correlation analysis with ground truth data to validate DT authenticity, offering a clear and compelling visualization of the effectiveness of ANCHOR-Grid to distinguish legitimate representations from deep-fake threats. This Graphic User Interface (GUI), depicted in Figure 3, transforms complex data into an intuitive and actionable tool, underscoring the practical relevance of our approach to smart grid security.

### 4.3. Micorgrid Monitoring in the Microverse

The Microverse environment provides a dynamic virtual space to simulate and manage smart grid components in real-time. It allows DTs of physical assets to interact with grid operations’ physical and virtual representations. Monitoring a microgrid within the Microverse involves integrating DTs, real-time data streams, and sophisticated analytics to provide a comprehensive overview of microgrid performance. By implementing monitoring capabilities within the Microverse, operators can observe, optimize, and secure power generation, consumption, and distribution while maintaining grid stability and responding quickly to anomalies or attacks.

#### 4.3.1. Digital Twin Integration and Real-Time Data Acquisition

The first step in monitoring a microgrid in the Microverse involves creating DTs for all major microgrid components, such as solar panels, wind turbines, battery storage systems, and inverters. Each DT is a virtual representation that mirrors the state and operation of its physical counterpart in real time. High-resolution Phasor Measurement Units (PMUs) and distributed IoT endpoints (e.g., smart meters, dedicated ENF-capture sensors) continuously sample voltage, current, phase angle, power output, temperature, and ENF at up to 100 Hz. Measurements are published via Data-Distribution Service (DDS) middleware for guaranteed low-latency delivery and, in remote sites, over secure 4G/5G channels using MQTT/TCP. Edge gateways perform STFT-based ENF extraction, noise filtering, and payload compression before encrypting JSON packets. The cloud ingestion layer writes streams into MongoDB time-series collections, leveraging bucketing, compression, and indexes on device ID and timestamp to support high-throughput writes and sub-second query latency. This architecture sustains update intervals below 500 ms and scales horizontally to mirror live grid dynamics in DTs.

Real-time data acquisition is fundamental to the monitoring process. A data gateway or communication hub collects sensor data from microgrid components, processes them, and transmits them to the Microverse. Within the Microverse, the data are ingested into DTs, allowing operators to visually monitor system status through a user interface (UI). This user interface offers an interactive, graphical dashboard that shows all key metrics. The data collected are further stored in a centralized database to enable detailed analysis, pattern recognition, and historical trend comparisons, which are crucial for understanding grid dynamics and enhancing predictive capabilities.

#### 4.3.2. Operational Analysis and Real-Time Monitoring

Real-time monitoring in the Microverse allows the system to observe microgrid performance metrics and react quickly to operational issues. By continuously comparing live data from physical assets with pre-defined thresholds or setpoints, the system can detect performance deviations that indicate potential problems, such as overvoltage, equipment overload, or low battery levels. Suppose the monitoring system detects anomalies, such as a sudden drop in power output from a solar array or a battery malfunction. In that case, it can trigger alerts, prompting operators to investigate and take corrective action.

Moreover, the Microverse environment allows for automated anomaly detection using machine learning models. Algorithms such as Long Short Term Memory (LSTM) networks and autoencoders can be deployed within the Microverse to analyze incoming data streams from the microgrid DTs. These algorithms identify anomalies that may not be detectable using traditional threshold-based monitoring by learning standard behavior patterns and detecting deviations in real-time. Such automated anomaly detection is beneficial for identifying early warning signs of faults, thereby preventing failures that could compromise the microgrid’s stability.

DTs in the Microverse provide a dynamic platform for operational analysis, enabling precise monitoring of microgrid components such as solar panels, wind turbines, battery storage systems, and inverters. Each DT mirrors its physical counterpart by integrating real-time sensor data (e.g., voltage, current, power output). This continuous data stream ensures that DTs reflect instantaneous grid states, facilitating proactive management. For instance, a DT of an inverter monitors power conversion efficiency, detecting deviations (e.g., >5% below nominal) that signal potential faults. These deviations trigger automated alerts visualized in the Microverse’s GUI, displaying metrics like active and reactive power on a 3D dashboard. Each DT instance receives real-time telemetry via an API gateway, comparing live inputs against model-driven envelopes (e.g., ±2% voltage, ±0.03 Hz ENF) and setpoints. Deviations trigger immediate event flags in the Microverse dashboard. Embedded LSTM predictors and convolutional autoencoders, trained on historical data (ENF standard deviation ≈0.015 Hz over 60 s windows), flag anomalies when normalized errors exceed a threshold of 3, enabling sub-second automated or operator-guided corrective actions.

#### 4.3.3. Security Monitoring and Attack Detection

Cybersecurity is an essential aspect of monitoring in the context of smart grids. The Microverse environment enables continuous security monitoring by integrating ENF signals as a real-world anchor for authenticating data between physical assets and DTs. The ANCHOR-Grid framework helps detect potential cyberattacks by embedding ENF-based signatures in the data packets. For instance, if an attacker tries to inject Deepfake data into the grid, the Digital Twin can verify the ENF signature and detect inconsistencies, effectively identifying the attack.

Another critical aspect of security monitoring is network traffic analysis. The Microverse environment employs IDSs that analyze communication data between microgrid components and their DTs. These systems monitor for suspicious activities, such as unauthorized access attempts or strange data flows, and raise alerts if a potential attack is detected. Combining ENF-based signature verification with advanced network security monitoring, the Microverse ensures that microgrid operations are secure, reliable, and protected against cyber threats.

Security monitoring leverages DTs to safeguard microgrid integrity against cyber threats, particularly Deepfake attacks that manipulate virtual representations. Each DT embeds ENF signals, captured by PMUs, as a physical layer authenticator. The Microverse integrates these signals into a security framework, where DTs cross-validate data authenticity in real time. For instance, a DT of a substation compares its ENF signature against a reference database, detecting discrepancies indicative of falsified data (e.g., Deepfake load reports). The implementation employs an anomaly-detection algorithm, quantifying deviations using the score $A_{\mathrm{ENF}}\left(t\right)=\frac{|f\left(t\right)-\hat{f}\left(t\right)|}{\sigma_{f}}$, where f(t) is the observed ENF, $\hat{f}\left(t\right)$ is the predicted ENF from a time series model, and $\sigma_{f}=0.02$ Hz is the standard deviation [43]. Scores exceeding 3 (e.g., >0.06 Hz deviation) trigger alerts, visualized as red flags on the UE5 GUI alongside metrics like correlation coefficients (>0.8 for authentic DTs). Running on edge devices with <10 ms latency, this process ensures a rapid response. Using Hyperledger Fabric, blockchain-based logging records ENF signatures, preventing tampering with a transaction latency of  0.5 s [26]. DTs’ relevance stems from their ability to anchor virtual operations to physical grid dynamics, thwarting attacks like false data injection, thus maintaining trust in Metaverse-integrated grids.

Every data packet carries an ENF watermark validated by computing the Pearson correlation between client and server ENF streams (requiring ρ≥ 0.8). Packets failing correlation or cryptographic signature checks are quarantined and marked *Compromised*. Concurrently, a micro-DT IDS inspects MQTT/TCP traffic, flagging protocol violations, payload anomalies, or replay attempts via time-sensitive ENF-based nonces, and correlates these with ENF alerts to distinguish benign network events from Deepfake or replay attacks. Compromised streams are isolated, and alerts are raised in real-time, ensuring only authenticated, integrity-verified data drives the virtual smart grid model.

## 5. Experimental Study

### 5.1. Experimental Setup

Based on the proof-of-concept prototype system in the Microverse environment, we validated our ANCHOR-Grid framework’s feasibility in detecting and mitigating security threats. We developed multiple simulations to test attack scenarios targeting data flows between physical devices and their respective DTs. Deepfake data-injection attacks involved introducing falsified sensor data into the grid, in an attempt to disrupt DT operations. To validate the authenticity of data packets, we introduced ANCHOR-Grid’s ENF-based signature mechanism.

#### 5.1.1. Physical Testbed

**Figure 4:**
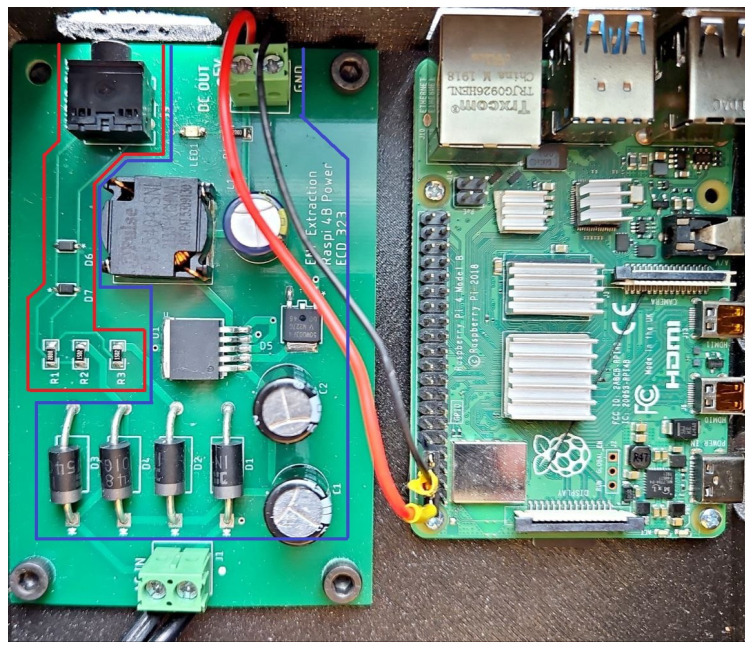
Physical device to extract ENF and test ANCHOR-Grid framework.

The ENF extraction and security-analysis testbed consists of two parts, which serve distinct but complementary purposes. The left printed circuit board (PCB) shown in Figure 4 is responsible for power regulation and signal conditioning, while the Raspberry Pi is the central processing and data-extraction unit. Together, these two boards provide a system capable of extracting ENF data from the power grid and processing it for security purposes.

The left PCB performs two critical functions. First, the blue section generates a regulated 5V DC power supply from the input voltage, which is then used to power the Raspberry Pi on the right. This regulated power is crucial for ensuring the stable operation of the Raspberry Pi, enabling it to process and extract ENF data efficiently. The red section of the left PCB is responsible for stepping down and conditioning the voltage from the power grid. This reduced voltage signal is then fed into the Raspberry Pi’s audio input. This section aims to provide a safe, scaled-down version of the AC power grid frequency, which can be captured and analyzed by the Raspberry Pi. The reduced voltage ensures that the Raspberry Pi receives only a low-amplitude representation of the power grid frequency. This makes it suitable for ENF extraction without posing a risk to the equipment or the operator. The Raspberry Pi, located on the right, serves as the central processing unit for the testbed. The audio input of the Raspberry Pi is used to capture the ENF signal, which represents variations in the frequency of the power grid. Once captured, the Raspberry Pi processes the streaming data to extract the ENF, which is used in the ANCHOR-Grid framework.

#### 5.1.2. ENF-Based Signature

The ANCHOR-Grid framework’s core is the use of ENF as an authentication anchor. The ENF signal fluctuates continuously depending on the grid conditions and provides a unique fingerprint for data packets originating within the power grid. To generate an ENF-based signature, we employed the following steps:

- Extracting ENF Data Window: A window of ENF data is extracted from the power grid. The specific security requirements determine the length of the window—typically, a 10-s window is used, during which the ENF value is sampled every second. This yields a sequence of ENF values, e.g., [60.01, 59.98, 60.02, 59.99, 59.97, …].
- Normalization of the ENF Data: To prepare the data for signature generation, min-max normalization is applied to scale the ENF values between 0 and 1. This helps in maintaining consistency across different environments. For instance, if $min_{val}$ is 59.96 and $max_{val}$ is 60.03, each value in the sequence is normalized as:
- Smoothing the ENF Data: Given that ENF data can be noisy, a moving average technique is used to smooth the sequence. This removes minor fluctuations, making the resulting signature more robust. Using a 3-point moving average, the smoothed sequence might look like [0.428, 0.619, 0.524, *…*].
- Hashing to Generate a Fixed-Length Signature: The smoothed ENF sequence is concatenated into a single string and then hashed using a cryptographic hash function, such as SHA-384, to generate a fixed-length ENF signature. This signature acts as a watermark that ties the data to real-world conditions in the grid. For instance, the hash output might look like: ENFSignature=“106eb0a4d3ce1da3828693ea6f364159a15c3bf3f637cb918ffe3db8e875b1948aff0ca9a1e6ce5cefc401a64302e3db”
- Generating data packet structure with JSON format typically includes metadata such as packet ID, device ID, timestamp, and the data payload (e.g., sensor readings). The structure of the data packet is as follows:
  - Packet ID: A unique identifier, e.g., “P164205785600”.
  - Device ID: The identifier for the originating device, e.g., “Device01”.
  - Timestamp: When the packet was generated, e.g., “2024-11-11T12:30:45Z”.
  - Data Payload: Sensor readings or measurements, e.g., {“temperature”: 25.4, “power_usage”: 12.5}.
  Once the data packet is generated, it is serialized to a JSON string and hashed using a cryptographic hash function, such as SHA-384, to produce a fixed-length message. Afterward, the ENF signature is combined with the hashed packet to form the final message. For the combination process, three approaches were followed:
  - Concatenation: Concatenate the two hashed values, deciding the order based on the timestamp. For example, if the timestamp is even, $Combined_{H}ash=Hash1\;\left|\right|\;Hash2$; otherwise, $Combined_{H}ash=Hash2\;\left|\right|\;Hash1$.
  - Interleaving: Use an empty list to store the combined result. Generate the message by iterating through each bit or byte of *Hash1* and *Hash2*, appending them according to the interleaving rule determined by the timestamp. For example, if the timestamp is even, start by appending a byte from *Hash1*, followed by a byte from *Hash2*, and repeat.
  - Pseudorandom Number Generator (PRNG): SHA-384 produces a hash of 48 bytes (or 96 hexadecimal characters). First, we generate a seed number based on the milliseconds of time we used in the packet to create 48 fixed random numbers within the range [0, 95]. These numbers are then used to place Hash1 and Hash2 in a 96-byte vector, which is subsequently sent to the server as a message.
  At first, we applied each approach independently. Then, to improve the randomness of CombinedHash, we employed a sequence of random combinations, utilizing different methods to create the message. This combination is derived from five approaches: two strategies for concatenation, two for interleaving, and one using the PRNG. One of these approaches is randomly selected (based on the seed number used in the PRNG) to generate the final message. Then, the final message is hashed and encapsulated in a JSON packet for transmission to the server.

**Figure 5:**
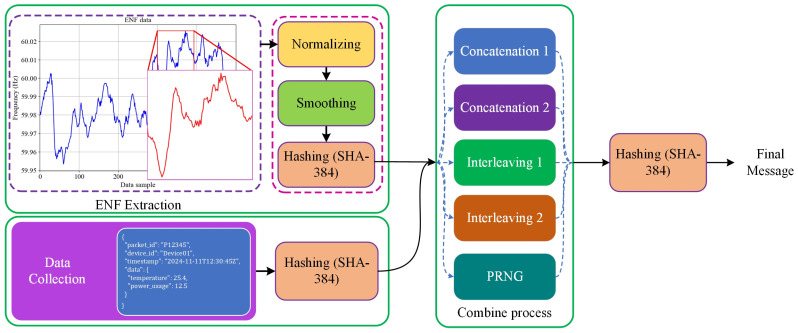
Hashing process of data with integration of ENF data.

After generating the final JSON message, AES-512 encryption was applied to the package to improve data security. Figure 5 illustrates how the ENF data is extracted, hashed, and combined with the message data. The body of the message contains the message data, which are as follows:

{

"message":

{

"packet_id": "P164205785600",

"device_id": "dev_102",

"date_time": "2024-11-11T12:30:45Z"

"data_payload": {

"temperature": 25.4,

"power_usage": 12.5

}

"enf_data": [60.01, 59.98, 60.02,

59.99, 59.97, …]

}

"signature": 8c7f1e1e606a22d3062f61d7f373

6836ee46ab4b32f49fbaedfe29c21969c7b924507

05e0a5643ae3aaae783cc3482d2

}

A timestamp is included in the packet to add another layer of security. This timestamp is key, to ensure that each packet is unique and time-bound. The inclusion of timestamps makes it difficult for attackers to replicate packets, as they would need precise access to the ENF data at a specific time to generate the correct packet. The combination process, which can use concatenation, interleaving, or PRNG-based combination, is selected based on the timestamp as the key.

#### 5.1.3. Evaluation Model

This study explores potential Deepfake attacks on the ANCHOR-Grid framework, an authentication method for data packets originating from the power grid that uses ENF signals as an authentication anchor. We assume that an attacker possesses detailed system knowledge, including public and private keys, but lacks information about the specific ENF signature embedded in each message. This paper outlines approaches to implement Deepfake attacks targeting different stages of the authentication process and replay attacks that use previously captured ENF signatures, emphasizing the generation of convincing ENF signatures and examining the robustness of the signature-generation process. Tampering attacks, where specific attributes of the packet (such as single bytes, timestamps, or payloads) are altered, also effectively test the ability of the mechanism to detect integrity violations. Furthermore, noise-injection attacks, which introduce random noise or jitter, can be used to assess the robustness of the generation and verification of ENF signatures. Finally, we evaluate the success rate of these fake attacks and their impact on the system’s reliability.

The evaluation model assumes an adversary that tries to bypass the authentication system using forged data packets. However, the primary focus is on assessing the success rate of the ENF-based authentication mechanism in distinguishing between legitimate and forged packets under different scenarios. Several attacks were used to examine the performance of the security algorithm in testing Deepfake attacks. Application Programming Interfaces (APIs) were used to receive data from the client and store it on the server.

#### 5.1.4. Methodology

A structured and repeatable evaluation methodology is essential for testing the performance of an ANCHOR-Grid defense mechanism. This methodology should encompass controlled experiments and, where feasible, simulations or real-world testing. A comprehensive testing approach helps ensure the defense mechanism is effective in various scenarios and performs well under varying conditions. In the following, we outline the key steps and considerations that guide this evaluation process.

We evaluated ANCHOR-Grid and alternative authentication mechanisms based on the following criteria to ensure meaningful and authentication-relevant comparison:

1. Resilience to Replay Attacks: Replay attacks involve capturing previously valid authentication data and reusing it to gain unauthorized access. Many cryptographic methods (e.g., hash-based authentication) mitigate replay attacks using timestamps and challenge-response mechanisms, but they can still be vulnerable to timestamp manipulation or clock synchronization issues. Physical layer authentication techniques (e.g., RF fingerprinting) generally resist replay attacks but require environmental stability to remain effective. ANCHOR-Grid, however, ensures replay resistance by leveraging real-time ENF fluctuations, which cannot be accurately predicted or replicated outside the physical grid environment.
2. Real-Time Performance and Scalability: Authentication mechanisms should operate with minimal latency, especially in real-time systems like smart grids. Cryptographic authentication methods require computational resources for encryption/decryption, which can introduce delays, particularly in resource-constrained IoT devices. Physical layer authentication (e.g., RF fingerprinting) can provide real-time responses but requires specialized signal processing hardware. Sensor-based authentication may involve data aggregation from multiple sources, leading to higher latency. In contrast, ANCHOR-Grid is designed for lightweight, real-time authentication, as ENF extraction and correlation require minimal processing power, enabling instantaneous authentication in distributed environments.
3. Adaptability to Dynamic Grid Environments: The smart grid infrastructure is highly dynamic, and the components continuously join and leave the network. Authentication mechanisms must be adaptive to changes in network topology and variable environmental conditions. Cryptographic approaches often require centralized key distribution, making them less flexible in large-scale, decentralized networks. Physical layer authentication methods can be susceptible to environmental variations, requiring frequent recalibration. Sensor-based methods rely on stable environmental conditions, limiting their adaptability. However, ANCHOR-Grid naturally adapts to dynamic environments, since ENF signals are inherently synchronized throughout the power grid, eliminating the need for manual adjustments.

Step 1: Defining Clear Metrics and Objectives

The first step in testing a defense mechanism is to define clear performance metrics and objectives. The metrics used to evaluate the effectiveness of the defense mechanism include the detection rate, the false positive rate (FPR), the false negative rate (FNR), and overall precision. The detection rate measures the percentage of successfully identified attacks or anomalies, while the FPR captures the proportion of benign activities mistakenly flagged as attacks. These metrics provide insight into the sensitivity and specificity of the defense mechanism, which is crucial to understanding its reliability. Additional performance measures include precision and recall, which provide further details about classification accuracy. Precision refers to the proportion of true positives among all flagged instances, while recall measures the ability to identify all actual attacks.

Step 2: Establishing a Baseline

Before implementing the defense mechanism, it is critical to establish a baseline measurement of system performance without any defensive measures. Baseline measurements allow for an objective comparison of performance changes post-implementation. These baselines may include metrics such as average packet processing time, memory usage, and overall system throughput in the absence of any security intervention. By comparing the system’s behavior before and after the defense mechanism is introduced, it becomes easier to quantify the actual impact of the new mechanism.

Step 3: Creating a Diverse Test Dataset

A key aspect of performance testing is using a diverse and comprehensive test dataset. The dataset should include legitimate traffic and various attack scenarios to thoroughly test the capabilities of the defense mechanism. Legitimate traffic consists of standard data packets that have not been altered, which helps evaluate the system’s ability to distinguish between benign and malicious activities. For a more robust evaluation, diverse attack scenarios should be included. These may involve Deepfake attacks, where modified data packets or ENF signatures mimic legitimate signals without originating from actual sources. The dataset should also vary in size and complexity, ranging from small datasets with just a few packets to large datasets. This allows the system’s scalability to be effectively evaluated. The complexity can be increased by varying the number of sensors, the variety of ENF signals, and environmental differences, providing a broader understanding of how well the defense mechanism performs in different contexts.

Generating a dataset of legitimate and altered packets requires following the specified rules. This dataset-creation process requires proper structuring of each component, from developing the ENF signature to crafting legitimate or forged packets, allowing for practical evaluation of the defense mechanism. This simulation aims to include correct and altered packets to evaluate the system’s ability to differentiate between them. The process starts with the generation of ENF data. Generated ENF data are crucial to simulate the real-world scenarios that the defense mechanism will encounter. We then normalize these data using Min-Max normalization, which scales the values between 0 and 1, ensuring uniformity before hashing. A moving average filter is applied to further refine the data to smooth out any abrupt variations and make the signature-generation process more reliable.

Once the ENF data are normalized and smoothed, the next step is to generate a signature using a cryptographic hashing algorithm. In this case, the SHA-384 hashing function generates a unique ENF signature. This signature acts as the identity of the ENF data and is crucial for differentiating between legitimate and altered packets. Following this, data packets are created by combining the ENF signature with a hashed version of the actual message. This combination is essential to ensure the authenticity of the packet. The legitimate packets are created using the correct message hash and the ENF signature.

In contrast, altered packets use a manipulated message, such as reversing the original message content before hashing. This alteration simulates an attacker trying to forge legitimate packets. The dataset is created in a structured format, with each packet accompanied by a label indicating whether it is legitimate or forged. The dataset is then saved in a CSV file for further analysis. The ENF correlation approach is integrated into the testing phase to validate the client and server ENF data similarity. This ensures that the defense mechanism can handle real-world noise and discrepancies effectively.

Step 4: Performing Controlled Experiments

Controlled experiments are an essential step in evaluating the defense mechanism’s performance. In a laboratory setting, known attacks can be injected at specified times, enabling precise measurement of how effectively and quickly the mechanism detects these threats. This type of experiment can also involve varying the volume and intensity of attacks, such as introducing a single altered packet rather than a large burst of modified packets. Repeating these experiments multiple times and averaging the results ensures statistical reliability and consistency.

A/B testing is another valuable tool for controlled experimentation. The effectiveness of the intervention can be demonstrated by comparing system performance with and without the defense mechanism under identical conditions. Stress and load testing further contribute to a complete assessment, as they evaluate how the defense mechanism scales when faced with increased traffic volume and attack frequency and determine the point at which the system may start to fail or experience significant slowdowns.

#### 5.1.5. Evaluation

The evaluation process starts with the system receiving data from a client, which contains two critical components: Signature and Message. These components verify the authenticity of the received data by cross-checking both the ENF and the cryptographic signature derived from the message content. The time parameter obtains the *Server ENF*, the ENF signature available on the server side for a specific time window. At the same time, the Client ENF is extracted from the data received from the client. This *Client ENF* is essentially the signature generated on the client side during the given time frame, using the ENF values that align with the time window of the data creation.

**Figure 6:**
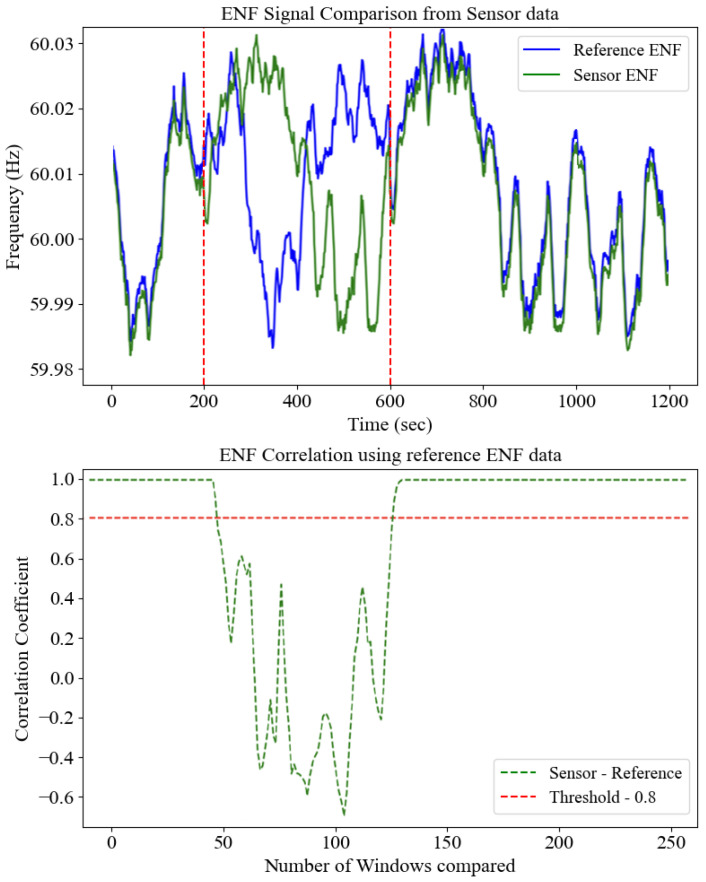
Correlation between the server and the client ENF.

The next step involves generating a Calculated Signature from the Message. The message is processed using cryptographic hashing (SHA-384), resulting in a unique hash value, the *Calculated Signature*. This calculated value is critical for verifying the integrity of the received message by ensuring that the data have not been altered during transmission. The Client Signature, included in the received packet, must match this calculated value for the data to be considered genuine. Once both the Server ENF and Client ENF are available, a correlation check is performed to determine the similarity between the server-side and client-side ENF data. Due to potential noise and variations during the ENF extraction process, the values may not be identical. Therefore, a threshold correlation value of 0.8 is established. If the correlation is more significant than 0.8, the server concludes that the client and server ENF data are sufficiently similar, allowing the system to proceed to the next validation step. However, if the correlation is below 0.8, the data are immediately flagged as fake since the ENF values are too different, indicating potential tampering or an attempted attack. Figure 6 shows the correlation process for detecting legitimate and fake ENF.

In the upper panel of Figure 6, the blue trace depicts the “ground-truth” ENF profile obtained from a high-precision phasor measurement unit (PMU). In contrast, the green trace shows the ENF extracted by our prototype sensor (using STFT and a 3-point moving average). Two vertical red dashed lines mark the start and end of a deliberate noise/spoof interval: outside this window, the sensor faithfully tracks the reference, but within it, the green and blue curves visibly diverge, illustrating compromised signal integrity. The lower panel translates these time-domain discrepancies into a sliding-window correlation analysis. We partition the 1200 s record into 250 equal-length segments and compute the Pearson correlation coefficient ρ between sensor and reference within each. The green dashed curve shows ρ versus window index, and the horizontal red dashed line at ρ = 0.8 indicates our decision threshold for authenticity. During genuine operation (approximately windows 1–50 and 121–250), ρ remains above 0.9, reflecting excellent agreement despite minor extraction jitter. In contrast, during the noise/spoof interval (windows 51–120), correlations plunge well below 0.8—and even become negative—signaling a loss of genuine ENF anchoring. This clear bimodal separation validates 0.8 as a practical cutoff: it tolerates normal sensor noise while reliably rejecting any Deepfake or replayed ENF segments.

After successfully passing the correlation check, the system compares the Client Signature (the hash signature provided by the client) with the Calculated Signature (derived from the message data at the server). If both signatures match, it confirms that the message has not been altered, and the data are validated further. On the other hand, if the signatures do not match, the data are labeled as Fake Data, signifying that the integrity of the message has been compromised. The final step in the evaluation process involves combining the outcomes of the correlation check and the signature verification. If both conditions are satisfied—the ENF correlation exceeds 0.8, and the Client Signature matches the Calculated Signature—the data are classified as Valid Data. This indicates that the message is authentic, and its temporal signature (the ENF) and integrity (the message signature) are intact. However, if either check fails, the data are labeled as Fake, ensuring that only authentic and unaltered data are accepted.

**Figure 7:**
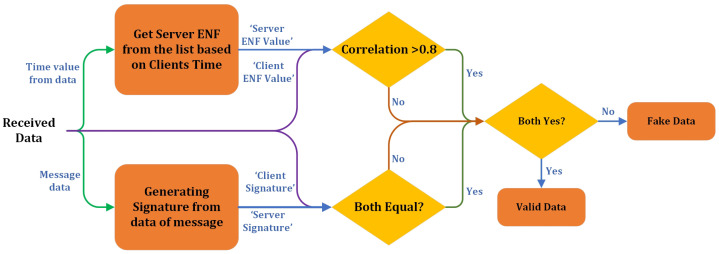
Evaluation process algorithm on the server side.

This multi-layered approach provides a robust mechanism for verifying data authenticity by combining temporal correlation and message integrity checks. Using both ENF correlation and cryptographic signatures adds multiple layers of security, making it exceedingly difficult for attackers to forge data successfully without access to the specific ENF values and the correct message signature. Thus, the system ensures data’s temporal alignment and integrity before marking them as valid. Figure 7 illustrates the evaluation process on the server side.

### 5.2. Experimental Results

#### 5.2.1. Detection Rates and False Positives

**Figure 8:**
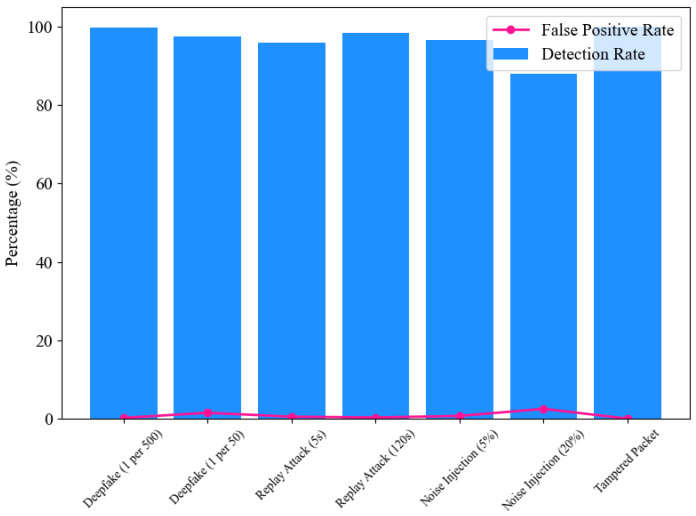
Detection rate and false positives under attack scenarios.

To evaluate the detection capabilities and robustness of the ENF-based defense mechanism, a series of tests were conducted under various attack scenarios with varying levels of complexity and frequency, as shown in Figure 8. For Deepfake attacks, the system was tested at low attack frequencies (one forged packet per 500 legitimate ones) and higher frequencies (one per 50). At lower frequencies, the mechanism achieved near-perfect detection (99.8%) with negligible false positives (0.2%), showcasing its sensitivity to sparse attack patterns. However, as the attack frequency increased, the FPR rose slightly to 1.5%, although the detection rate remained robust at 97.5% as shown in Table 1.

Replay attacks were evaluated with different time offsets to measure the system’s resistance to temporal manipulation. ENF signatures reused after a short delay (5 s) were more challenging to detect, resulting in a slightly lower detection rate of 94%. However, more prolonged delay attacks (120 s) were effectively mitigated, with a detection rate of 98.5%, highlighting the system’s temporal resilience. Tampered data packets were also tested, including scenarios where multiple bytes in the payload were modified. These tests consistently demonstrated perfect detection rates (100%), with no false positives. Random variations were introduced into the ENF data to assess the system’s robustness against noise. While minor noise levels (5%) had minimal impact on detection (96.5% rate), significant noise (20%) reduced accuracy to 88%, indicating the need for enhanced robustness under high-noise conditions.

#### 5.2.2. Robustness Under Network Conditions

**Figure 9:**
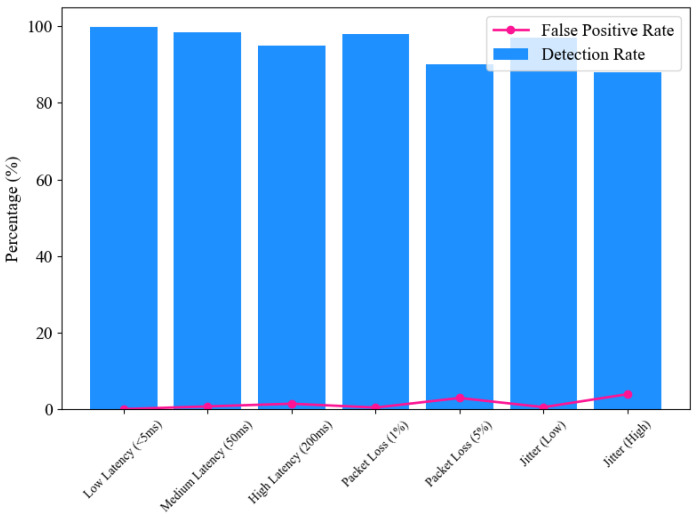
Performance of ANCHOR-Grid under network conditions.

The defense mechanism’s performance was further evaluated under various simulated network conditions, including latency, packet loss, and jitter, illustrated in Figure 9. In low-latency environments (<5 ms), the system exhibited near-perfect detection accuracy (99.9%) and minimal false positives (0.1%). As latency increased to 50ms and 200ms, detection rates slightly decreased to 98.5% and 95%, respectively, while false positives increased modestly, indicating some sensitivity to delayed network conditions.

Table 2 shows packet loss scenarios simulated real-world challenges in data-transmission reliability. At 1% packet loss, the system maintained a high detection rate (98%) with minimal impact on latency and false positives. However, with 5% packet loss, detection accuracy dropped to 90%, and false positives increased to 3%, revealing limitations under significant data loss. The system’s resilience to timing variations was assessed through jitter simulations. Low jitter conditions had negligible impact, maintaining a detection rate of 97%. In contrast, high jitter significantly affected performance, reducing detection accuracy to 88% and increasing false positives to 4%. These results underscore the importance of optimizing the system for real-world network environments.

#### 5.2.3. Comparison Between ANCHOR-Grid and Existing Security Mechanisms

We have conducted a detailed comparison study between ANCHOR-Grid and existing security mechanisms. Table 3 summarizes the key features concerning modern ESS optimization and security approaches, such as Cryptographic Signatures, Threshold-Based Anomaly Detection, AES Encryption, ECC, and IDSs, highlights significant differences in adaptability, robustness, scalability, and suitability for modern smart grid environments.

The core authentication methods for these mechanisms vary widely. ANCHOR-Grid uniquely uses ENF signals as real-world environmental fingerprints, embedding physical characteristics into the digital system. This approach inherently ties the data’s authenticity to the network’s physical state, offering a novel layer of security. In contrast, cryptographic signatures rely on hashing algorithms like SHA-384 to ensure data integrity, but they are static and lack adaptability. Threshold-Based Anomaly Detection focuses on predefined limits for parameters like voltage or frequency, while AES encryption secures data payloads without addressing authenticity. ECC provides secure key exchange and signing capabilities, but requires significant computational resources. An IDS monitors network traffic patterns, but its effectiveness is limited to predefined attack signatures.

Regarding adaptability, ANCHOR-Grid outshines traditional mechanisms by dynamically authenticating data and adapting to evolving threats like Deepfake and replay attacks. Conventional approaches are rigid and often fail against advanced threats. For example, Cryptographic Signatures and AES depend on static configurations, making them vulnerable to replay and adaptive attacks. Similarly, Threshold-Based Detection and IDSs rely on predefined patterns or rules, which struggle to address novel cyber threats. Robustness Against Deepfake Attacks is a critical advantage of ANCHOR-Grid. Using ENF signals, the ANCHOR-Grid detects and differentiates between authentic and fake data streams, which is a challenge for traditional methods. Cryptographic Signatures and AES Encryption are ineffective against Deepfake attacks, as these mechanisms cannot detect manipulated data if the payload structure remains intact. An IDS is also limited in handling legitimately mimicked behavior, making it less effective in addressing Deepfake scenarios.

Replay Attack Resilience is another area where ANCHOR-Grid excels. By analyzing the temporal properties of ENF signals, it identifies reused data from different time frames, effectively mitigating replay attacks. In contrast, Cryptographic Signatures and ECC can implement timestamping but remain vulnerable to spoofing. AES and IDSs lack inherent replay attack protection unless combined with external mechanisms. The noise resilience of ANCHOR-Grid is notable, as it maintains high detection accuracy (>85%) under moderate noise levels by filtering fluctuations in ENF signals. Traditional mechanisms like Threshold-Based Detection are highly susceptible to noise, which skews their predefined values, leading to false positives or missed anomalies. Although Cryptographic Signatures, AES, and ECC are not affected by noise in their operation, they fail to address anomalies caused by environmental noise. At the same time, IDSs may experience degradation in detection under noisy conditions.

Regarding real-time detection, ANCHOR-Grid supports decentralized, lightweight, real-time authentication, which is ideal for distributed smart grid environments. Existing methods like Cryptographic Signatures and ECC are not designed for real-time responses. At the same time, Threshold-Based Detection and IDSs provide real-time monitoring but lack ANCHOR-Grid’s adaptability and scalability. Scalability is another significant strength of ANCHOR-Grid. Its decentralized use of ENF signals allows it to scale seamlessly across large smart grid systems. In contrast, traditional mechanisms such as ECC and IDSs face scalability challenges due to key-management and traffic-analysis requirements. Although cryptographic signatures and AES scale reasonably well, they still require centralized management for complex deployments.

The implementation complexity for ANCHOR-Grid is moderate, requiring ENF signal extraction, but it avoids the heavy cryptographic dependencies seen in ECC and IDSs. Cryptographic Signatures and Threshold-Based Detection are more straightforward to implement but lack the effectiveness needed for modern threats. ECC and IDSs, on the other hand, are more complex due to the need for cryptographic operations and extensive network monitoring. When integrated with IoT devices, ANCHOR-Grid’s lightweight ENF-based approach makes it an ideal choice, particularly for devices with limited space in smart grid environments. Traditional mechanisms like Cryptographic Signatures and Threshold-Based Detection are compatible with IoT but lack dynamic protection. ECC and IDSs are resource-intensive, which limits their practicality for IoT systems. Lastly, the primary limitations of these mechanisms reveal the edge of the ANCHOR-Grid in modern applications. Although ANCHOR-Grid is sensitive to extreme noise levels (>20%), which can reduce its accuracy, traditional methods struggle against novel adaptive attacks. Cryptographic signatures are vulnerable to replay and key theft, Threshold-Based Detection fails against sophisticated threats, and ECC and IDSs are resource-heavy and less effective for IoT systems.

#### 5.2.4. Comparison Between ANCHOR-Grid and Other Authentication-Related Approaches

Encryption (e.g., AES, ECC) and IDSs play essential roles in cybersecurity. However, they are primarily designed for data confidentiality and network monitoring, rather than device authentication. We conducted a detailed comparison study between ANCHOR-Grid and other authentication methods that address similar challenges in the security of smart grid infrastructure. Table 4 shows a detailed comparison between other authentication methods.

1. Cryptographic-Based Authentication (e.g., Elliptic Curve Cryptography, Hash-Based Authentication): Cryptographic-based authentication methods, such as Elliptic Curve Cryptography (ECC) and Hash-Based Authentication, rely on digital signatures, cryptographic hashes, and asymmetric encryption to verify identities. These methods provide strong authentication against unauthorized access and manipulation. However, they depend on pre-shared keys or certificates, introducing vulnerabilities like key compromise, certificate spoofing, and replay attacks. ANCHOR-Grid, in contrast, eliminates reliance on static cryptographic keys by using real-time ENF signals as a dynamic, naturally occurring authentication factor, making it more resilient to key-based attacks.
2. Physical Layer Authentication (e.g., RF Fingerprinting, Power Side-Channel Authentication): Physical layer authentication techniques use hardware-specific characteristics such as RF signal variations, device-specific power-consumption patterns, or side-channel emissions to verify the device’s authenticity. RF fingerprinting uniquely identifies a device based on its wireless transmission characteristics, while power-side channel-authentication analyzes energy-consumption patterns. These methods offer strong resistance to software-based cloning and impersonation attacks. However, their effectiveness depends on specialized hardware requirements and environmental stability (e.g., RF signals can fluctuate due to interference). ANCHOR-Grid provides a more scalable and grid-wide authentication approach, leveraging the inherent ENF signal consistency across interconnected power systems rather than requiring individual device calibration.
3. Sensor-Based Authentication (e.g., Multi-Sensor Fusion, Biometric-Inspired Approaches): Sensor-based authentication methods use multimodal sensor inputs to create unique, location-dependent authentication factors. These approaches have been explored in biometric authentication, industrial IoT security, and environmental monitoring. Although effective in certain applications, they often require continuous sensor calibration, suffer from drift over time, and may be vulnerable to sensor spoofing. ANCHOR-Grid leverages the intrinsic, non-replicable nature of real-time ENF variations, making it more resistant to sensor-based spoofing while requiring no additional hardware beyond grid-connected measurement devices.

**Table 4:** Comparison of authentication-related approaches.

Feature	ANCHOR-Grid (ENF-Based)	Cryptographic Authentication (ECC, Hash-Based) [63]	Physical Layer Authentication (RF Fingerprinting, Power Side-Channel) [64]	Sensor-Based Authentication (Multi-Sensor Fusion, Biometric-Inspired) [65]
Core Authentication Method	Uses real-time ENF signals as environmental fingerprints to authenticate grid components.	Uses cryptographic keys and digital signatures for verification.	Utilizes hardware-specific properties like RF emissions or power usage.	It relies on sensor data to create authentication patterns.
Replay Attack Resilience	High—ENF signals are time-variant and cannot be replayed.	Moderate—Timestamp-based protection, but vulnerable to clock manipulation.	High—Unique physical properties are difficult to replicate.	Low—Sensor data can be captured and replayed.
Real-Time Performance	High—ENF extraction and correlation require minimal processing power.	Moderate—Encryption/decryption can introduce computational delays.	High—RF and power-based authentication occur in real-time.	Low—Requires data aggregation and processing from multiple sensors.
Scalability	High—No pre-shared keys; applies naturally to grid-wide systems.	Low—Key distribution and management challenges in large networks.	Low—Requires specialized hardware and calibration per device.	Moderate—Scales with sensor networks but increases computational cost.
Adaptability to Dynamic Environments	High—ENF signals are naturally synchronized across the power grid.	Low—Requires centralized key management and updates.	Moderate—Environmental factors may impact reliability.	Low—Sensor drift and environmental changes affect accuracy.
Hardware Dependency	Low—Uses existing power grid infrastructure; no additional hardware required.	High—Requires cryptographic modules and key storage.	High—Needs specialized RF or power monitoring hardware.	High—Requires deployment of multiple sensors per authentication node.
Resistance to Deepfake Attacks	High—ENF signals cannot be artificially generated or Deepfaked.	Low—Deepfake AI can generate fraudulent cryptographic responses.	High—Device-specific RF/power signatures are hard to fake.	Low—AI can generate synthetic sensor data to mimic real-world readings.
Tamper Detection	High—Blockchain-based ENF verification prevents data manipulation.	Moderate—Digital signatures protect against tampering but depend on key security.	High—Physical fingerprinting makes tampering difficult.	Low—Sensor readings can be manipulated without detection.
Computational Overhead	Low—ENF extraction and correlation are lightweight.	High—cryptographic operations require computational resources.	Moderate—Requires real-time RF/power signal processing.	High—Multi-sensor fusion demands continuous data processing.
Implementation Complexity	Moderate—Requires ENF extraction and blockchain integration.	High—Needs cryptographic key infrastructure and certificate management.	High—Demand specialized hardware for RF/power analysis.	High—Requires extensive sensor deployment and data-fusion mechanisms.
Security Against Cloning	High—ENF signatures are unique and cannot be replicated.	Low—Cryptographic keys can be stolen or cloned.	High—Hardware properties are unique to each device.	Low—Sensors can be spoofed or cloned.

## 6. Conclusions

This study introduces ANCHOR-Grid, an innovative method for securing smart grid DT by utilizing ENF signals as real-world anchors. As smart grids increasingly depend on DTs for real-time monitoring, optimization, and predictive analysis, the risks associated usingwith Deepfake attacks and data manipulation also increase. ANCHOR-Grid addresses these issues by embedding distinctive ENF-based environmental fingerprints within data streams, effectively differentiating legitimate and fraudulent inputs. This method guarantees that DTs’ integrity, authenticity, and reliability remain intact, even under complex adversarial conditions. Through extensive simulations, ANCHOR-Grid has shown its ability to detect and counter various cyber threats, including Deepfake and replay attacks, noise injection, and tampering. The framework’s capacity to maintain a high detection rate, even in challenging situations such as a higher attack frequency or network latency, highlights its robustness and flexibility. Furthermore, employing ENF signals as a temporal and spatial fingerprint provides a lightweight, cost-effective, and tamper-resistant security layer, minimizing reliance on traditional cryptographic or GPS-based methods that may be susceptible to spoofing or other attacks.

Beyond its technical contributions, ANCHOR-Grid signifies a paradigm shift in how physical and digital systems interact and authenticate within critical infrastructure. The framework bridges the gap between the physical and virtual realms by anchoring DTs to the variable real-world conditions of the physical grid, ensuring smooth integration and synchronization. This capability improves the resilience of smart grid systems and lays the groundwork for broader applications of environmental fingerprinting in other fields such as transportation, healthcare, and smart cities. The study also outlines significant directions for future research, including scaling the ANCHOR-Grid framework to accommodate larger and more complex infrastructures, integrating it with emerging technologies like blockchain for decentralized security, and investigating its applicability in scenarios beyond smart grids.

Furthermore, improving its resilience to high environmental noise levels and developing more sophisticated anomaly-detection algorithms can enhance its utility and robustness. ANCHOR-Grid exemplifies how real-world ecological signals can be harnessed to fortify digital ecosystems against evolving cyber threats. Its innovative design, validated by rigorous testing, safeguards smart grid operations and sets a precedent for integrating real-world anchors into digital infrastructures. This research contributes significantly to advancing secure, trustworthy, and resilient digital twin systems, paving the way for safer and more reliable smart grid ecosystems in an increasingly interconnected era.

## Figures and Tables

**Table 1:** Precision and recall in different attack scenarios.

Attack Type	Precision (%)		Recall (%)	
	Baseline	ANCHOR-Grid	Baseline	ANCHOR-Grid
Deepfake (1 per 500)	91	99.8	85	99.8
Deepfake (1 per 50)	88	98.4	80	97.5
Replay Attack (5 s old)	75	99.5	70	94
Replay Attack (120 s old)	85	99.7	80	98.5
Noise Injection (5% Noise)	78	99.2	70	96
Noise Injection (20% Noise)	65	97.1	60	85
Tampered Packet	90	100	85	100

**Table 2:** Robustness of ENF-based defense mechanism under network conditions.

Network Condition	Precision (%)	Recall (%)
Low Latency (<5 ms)	99.9	99.9
Medium Latency (50 ms)	99.2	98.5
High Latency (200 ms)	95.4	95
Packet Loss (1%)	99.5	98
Packet Loss (5%)	96.7	90
Jitter (Low)	99.4	97
Jitter (High)	95.6	88

**Table 3:** Comparison of the existing ESS optimization methods.

Feature	ANCHOR-Grid Framework	Cryptographic Signatures [16]	Threshold-Based Anomaly Detection [18]	AES Encryption [20]	ECC [9]	IDS [25]
Core Authentication Method	Uses ENF signals as environmental fingerprints.	Generates fixed-length signatures for data integrity.	Monitors specific parameters for anomalies.	Encrypts data payloads for confidentiality.	Provides secure key exchange and signing.	Detects attack patterns via traffic analysis.
Adaptability	Highly adaptable to dynamic, evolving threats like Deepfake and replay attacks.	Static; vulnerable to replay and adaptive attacks.	Ineffective against crafted or evolving threats.	Focused on encryption, not adaptability.	Limited to predefined patterns.	Struggles with novel and adaptive threats.
Robustness Against Deepfake	Differentiates fake data by leveraging ENF signals as anchors.	Vulnerable to fake data injection.	Ineffective; detects only gross anomalies.	Ineffective against data manipulation.	Cannot handle mimicked legitimate behavior.	Detects Deepfakes poorly unless explicitly trained for them.
Replay Attack Resilience	Detects replay attacks using temporal ENF correlations.	Timestamping helps, but spoofing is possible.	No inherent protection.	No inherent protection.	Limited unless integrated with timestamps.	May detect replay patterns via anomalies in traffic flow.
Noise Resilience	Maintains accuracy (>85%) under moderate noise.	Struggles as noise impacts static thresholds.	Ineffective as noise affects parameter detection.	Noise has no direct impact.	Moderate noise can degrade detection.	Performance degrades significantly if noise mimics legitimate traffic.
Real-Time Detection	Lightweight supports decentralized real-time detection.	Real-time but static in capability.	Real-time but limited to thresholds.	Not designed for real-time response.	Real-time but with heavy computation.	Real-time detection but computationally expensive at scale.
Computational Efficiency	Lightweight, scalable for IoT and distributed systems.	Moderate; computationally efficient.	Highly efficient for static thresholds.	Computationally intensive for IoT devices.	Computationally intensive at scale.	Heavy processing for real-time traffic analysis.
Scalability	Decentralized ENF signals enable scalability.	Scales well for simple setups.	Simple and scalable for static systems.	Less scalable due to key management.	Limited scalability for complex systems.	Requires substantial infrastructure for large-scale networks.
Implementation Complexity	Moderate; ENF signal extraction requires specialized hardware but avoids heavy cryptographic dependency.	Simple implementation; relies on hashing algorithms.	Simple but dependent on predefined values.	Complex due to cryptographic operations.	Complex; requires network traffic monitoring.	Implementation requires signature updates and frequent maintenance.
Integration with IoT Devices	Designed for lightweight IoT integration using ENF-based signatures.	Moderate; IoT-compatible hashing.	Easily deployable but lacks dynamic protection.	Requires resources unsuitable for IoT.	Resource-intensive for IoT systems.	Overhead limits practical IoT deployment without optimization.
Primary Limitation	Sensitive to extreme noise (>20%), reducing detection accuracy.	Vulnerable to replay attacks and stolen keys.	Fails against dynamic, adaptive threats.	Resource-heavy for constrained devices.	Ineffective for novel attacks.	Requires retraining for emerging attack vectors; labor-intensive.

## Data Availability

Data are contained within the article.

## Abbreviations

The following abbreviations are used in this manuscript:

APIs	Application Programming Interfaces
AI	Artificial Intelligence
DDDAS	Dynamic Data Driven Application Systems
DDoS	Distributed Denial-of-Service
DDS	Data-Distribution Service
DT	Digital Twin
DTs	Digital Twins
DoS	Denial-of-Service
ECC	Elliptic Curve Cryptography
ENF	Electric Network Frequency
ESS	Energy Storage Systems
FNR	False Negative Rate
FPR	False Positive Rate
GUI	Graphic User Interface
IoSGT	Internet of Smart Grid Things
IoT	Internet of Things
IDS	Intrusion-Detection System
LFC	Load Frequency Control
LSTM	Long Short Term Memory
MQTT	Message Queuing Telemetry Transport
PCB	Printed Circuit Board
PMUs	Phasor Measurement Units
PRNG	Pseudorandom Number Generator
SCADA	Supervisory Control and Data Acquisition
TCP	Transmission Control Protocol
UE5	Unreal Engine 5
